# Demonstration of 100 Gbps coherent free-space optical communications at LEO tracking rates

**DOI:** 10.1038/s41598-022-22027-0

**Published:** 2022-10-31

**Authors:** Shane M. Walsh, Skevos F. E. Karpathakis, Ayden S. McCann, Benjamin P. Dix-Matthews, Alex M. Frost, David R. Gozzard, Charles T. Gravestock, Sascha W. Schediwy

**Affiliations:** International Centre for Radio Astronomy Research, The University of Western Australia, Perth, 6009 Australia

**Keywords:** Adaptive optics, Fibre optics and optical communications

## Abstract

Free-space optical communications are poised to alleviate the data-flow bottleneck experienced by spacecraft as traditional radio frequencies reach their practical limit. While enabling orders-of-magnitude gains in data rates, optical signals impose much stricter pointing requirements and are strongly affected by atmospheric turbulence. Coherent detection methods, which capitalize fully on the available degrees of freedom to maximize data capacity, have the added complication of needing to couple the received signal into single-mode fiber. In this paper we present results from a coherent 1550 nm link across turbulent atmosphere between a deployable optical terminal and a drone-mounted retroreflector. Through 10 Hz machine vision optical tracking with nested 200 Hz tip/tilt adaptive optics stabilisation, we corrected for pointing errors and atmospheric turbulence to maintain robust single mode fiber coupling, resulting in an uninterrupted 100 Gbps optical data link while tracking at angular rates of up to 1.5 deg/s, equivalent to that of spacecraft in low earth orbit. With the greater data capacity of coherent communications and compatibility with extant fiber-based technologies being demonstrated across static links, ground-to-low earth orbit links of Terabits per second can ultimately be achieved with capable ground stations.

## Introduction

Communication at optical frequencies revolutionised terrestrial communications with the advent of optical fiber networks^1^, but the same is not yet true of free-space applications, which are still dominated by radio frequency (RF) communications. While the orders-of-magnitude increase in carrier frequency from RF (kHz–GHz) to optical frequencies (THz) enables a commensurate increase in data capacity, moving to the optical domain brings with it new challenges. Primary among these are the strict acquisition and tracking requirements^2^, and the effect of atmospheric turbulence that significantly influences optical beam propagation on millisecond timescales^3^. To realise the potential of free-space optical communications for ground-to-ground, ground-to-air, and ground-to-space links, the effects of atmospheric turbulence must be suppressed^4^.

The most straightforward implementations of free-space optical communications modulate data on the intensity of light, such as simple on-off keying (OOK) or pulse position modulation (PPM). These direct-detection methods only require a detector that can measure the intensity of the received light. Coherent detection methods, in contrast, maintain phase and polarisation information by mixing the received signal with a local oscillator (LO), giving extra degrees of freedom to encode data and capitalize fully on channel capacity^5^ and compatibility with ubiquitous fiber-based technologies^6^. These coherent methods require coupling the received light into single mode fiber (SMF), which at a diameter of 8–10 $\upmu$m, is more susceptible to pointing errors and turbulence compared with the larger multi-mode fiber ($>50\,\upmu$m) or free-space detectors used with direct-detection schemes^7^.

Currently, earth observation satellites produce data at such high volumes that on-board compression is often required before transmission to the ground using available RF bandwidth (e.g.^8,9^), which is power intensive and can reduce data fidelity. For the case of low earth orbit (LEO), the time a spacecraft is visible to any particular ground station is only a few minutes per day, further constraining data transfer. A LEO spacecraft could transmit data via a relay spacecraft, typically in geosynchronous earth orbit (GEO), but the increased transmission distance ($\sim \,35{,}000$ km versus $\sim \, 1000$ km) further burdens the size, weight, and power (SWaP) of spacecraft communications systems. Relieving this bottleneck is the goal of NASA’s Terabyte Infrared Delivery system (TBIRD) to develop cubesat-suitable optical terminals capable of 200 Gbps coherent LEO-to-ground downlink^10^.

The current 5.6 Gbps record for an optical data link between LEO to ground was demonstrated using coherent binary phase shift keying (BPSK) between two ESA TESAT laser communication terminals, one on board the NFIRE spacecraft and one on the ground at Tenerife, Spain^11^. These terminals were engineered for inter-satellite links, where atmospheric turbulence is not an issue and as such do not employ any active turbulence mitigation; only a reduction of the ground terminal aperture to reduce the effect of scintillation. The $\sim \,5$ m beam size would ensure that the occurrence of deep fades due to beam wander at the ground terminal are negligible, but given turbulence in a ground-to-space link is concentrated at the ground, beam wander is significantly greater for the uplink than the downlink. This is reflected in the disparity in link quality, with the downlink remaining error free while the uplink showed a bit-error rate (BER) of $\sim \,10^{-5}$, despite the identical hardware at each end. To push the data rates into the 100+ Gbps regime requires, at a minimum, tip/tilt adaptive optics (AO) stabilisation to improve downlink fiber coupling efficiency and pre-compensate uplink beam wander. Such ground stations are currently in development^12,13^ and have demonstrated AO-corrected SMF coupling from GEO^14^, but to our knowledge tip/tilt AO stabilised coupling has not been demonstrated at the more challenging tracking rates of LEO.

Tip/tilt AO stabilised high-speed coherent optical links between the ground and airborne platforms have been demonstrated previously. Chen et al.^15^ demonstrated a 100 Gbps bi-directional quadrature amplitude modulation (QAM) link between a ground station and light aircraft over 10–20 km link distance. Li et al.^16,17^ demonstrated an 80 Gbps link to a drone mounted retroreflector across a 100 m round trip distance with simulated turbulence, using two orbital-angular-momentum (OAM) multiplexed 40 Gbps quadrature phase shift keying (QPSK) links. These demonstrations reached angular tracking rates of ${\sim \,0.2}$ and ${\sim \,0.1}$ deg/s respectively, although maintaining fiber coupling at LEO-like tracking rates were not aims of those experiments.

The ultra-high capacity of coherent free-space optical communications has been demonstrated across static links by various groups. Parca et al.^18^ used 16 channel QPSK to establish a 1.6 Tbps link over 80 m between buildings. Feng et al.^19^ used 3 channel QPSK to achieve 160 Gbps over a 1 km link. The highest capacity link to date, by Docchan et al.^20^, achieved 13.16 Tbps with 54 channel QPSK with tip/tilt stabilisation across a turbulent 10.45 km link. Most recently, Guiomar et al. achieved the highest spectral efficiency to reach 800 Gbps in a single channel using probabilistic constellation shaping 64-ary QAM over 42 m.

To enable these high-capacity technologies for ground-to-space links requires a tracking system that can maintain SMF coupling in the presence of large angular velocities and atmospheric turbulence. In this paper, we present results of a coherent free-space optical link operating at 1550 nm between a deployable optical terminal and an airborne drone. Combining a tip/tilt AO system with concurrent closed loop machine vision (MV) tracking, we maintain the SMF coupled link at angular velocities up to ${\sim \, 1.5}$ deg/s, representative of the apparent motion of spacecraft in LEO.

**Figure 1:**
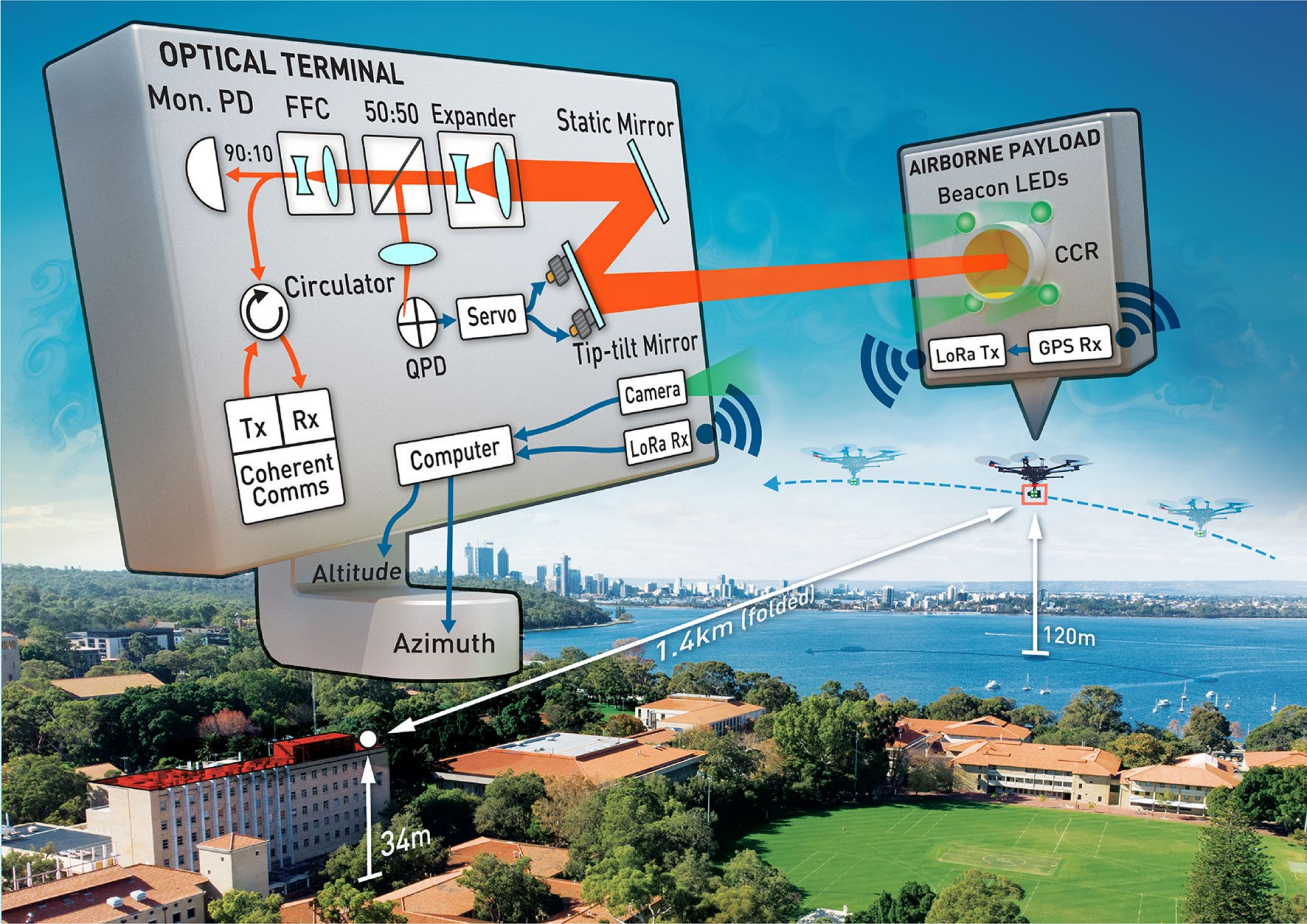
Schematic of the deployable optical terminal and experiment. *Mon. PD* monitoring photodetector, *FFC* fiber to free-space collimator, *QPD* quadrant photodetector, *CCR* corner-cube retroreflector, *LED* light emitting diode, *LoRa* “Long Range”, *Tx* transmitter, *Rx* receiver.

Our work uses the retroreflected signal serving as its own tip/tilt beacon^21,22^, which due to atmospheric reciprocity^23^ allows our terminal to simultaneously demonstrate correction of the “downlink” beam to maintain fiber coupling as well as pre-compensation of the “uplink” to maintain pointing on target. An overview of the experiment is depicted in Fig. 1. Our deployable optical terminal serves as a development test-bed for the Western Australian Optical Ground Station (WAOGS-1)^24^, and also as a standalone unit, which with further optimisation could facilitate Tbps ground-to-ground, ground-to-air, and ground-to-LEO coherent optical links.

## Methods

**Figure 2:**
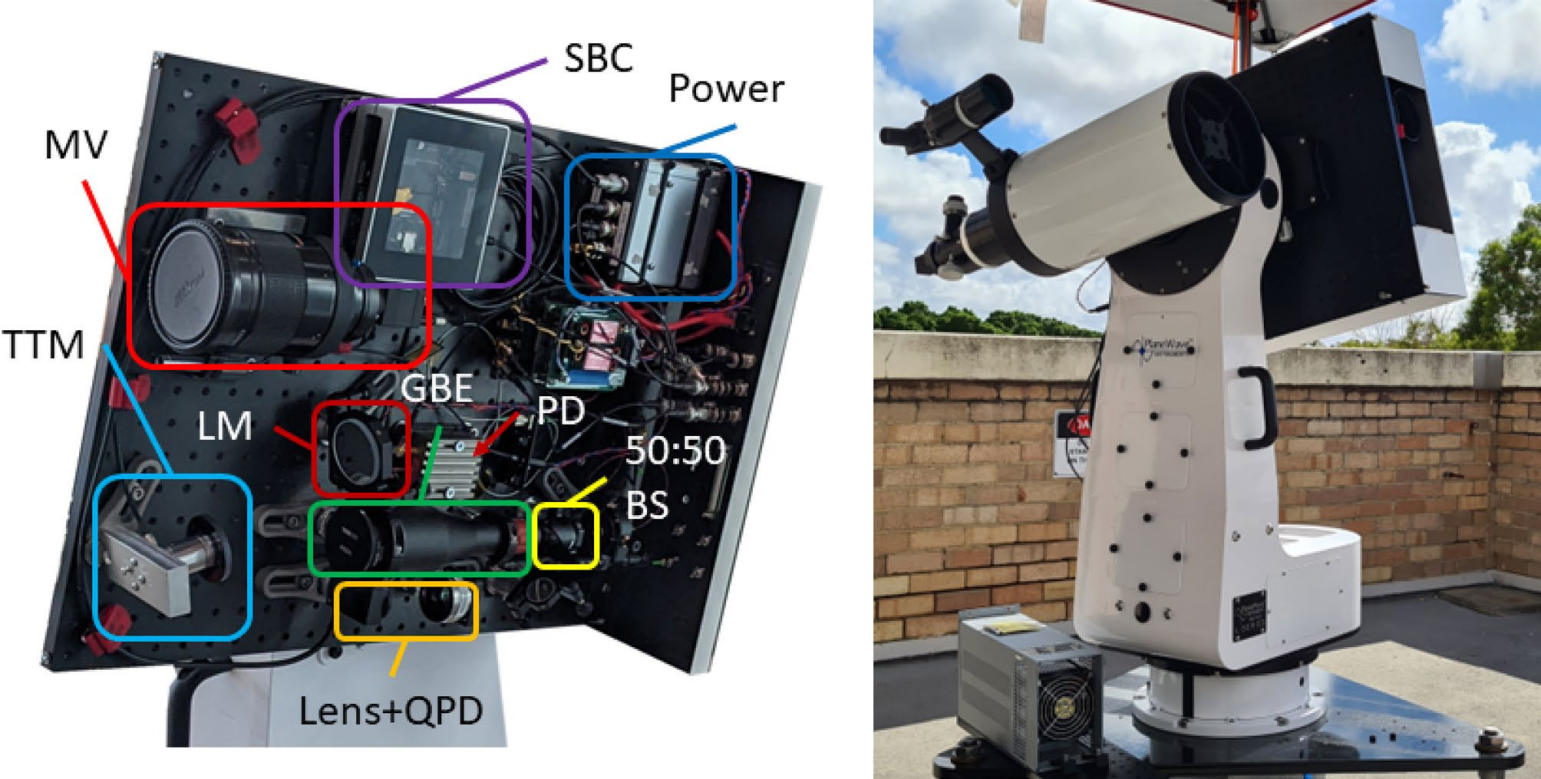
Left: the optical breadboard layout. *MV* Machine vision lens and camera, *TTM* tip/tilt mirror, *LM* static launch mirror, *GBE* Galilean beam expander, *QPD* quadrant photodetector, *SBC* single board computer, *PD* photodetector, *BS* beamsplitter. Right: the deployed optical terminal. Optics and MV are located on the far side of the breadboard. The tip/tilt control electronics are visible on the lower left of the mount base. The telescope mounted on the left was not used for this experiment.

**Figure 3:**
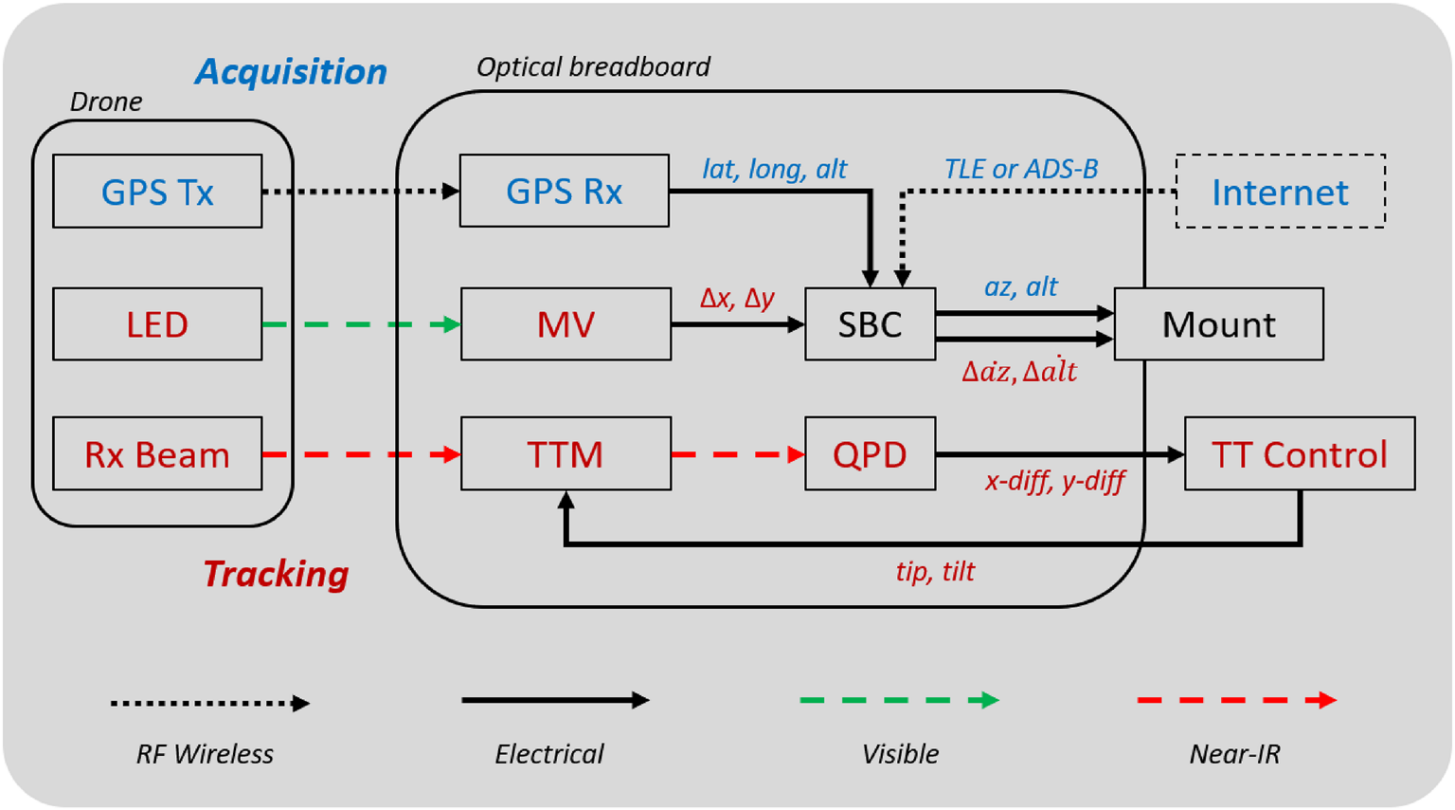
Block diagram showing interfaces between components during initial acquisition (blue) and continous tracking (red).

For this experiment our deployable optical terminal was located on the roof of the physics building at the University of Western Australia Crawley campus, approximately 34 m above sea level. An optical breadboard housing the optics, MV system, GPS receiver, and single board computer was fastened to the mount, shown in Fig. 2. Electrical cables and optical fiber carried signals to the tip/tilt AO control electronics on the mount base and communications equipment housed in a separate enclosure. To simulate a satellite pass, we used a drone carrying an optical payload that includes a corner-cube retroreflector (CCR), flying at an altitude of 120 m over the Swan River and a line-of-sight distance of 500–700 m for a folded link length of up to 1.4 km. Figure 3 details the interactions between components during the acquisition and tracking phases. CCRs installed at two fixed locations provided static links of 600 m and 2.4 km folded lengths, used for calibration and troubleshooting. A summary of the mount design parameters is presented in Table 1 and each subsystem is described in further detail in the following subsections.

**Table 1:** Mount parameters.

Parameter	Value
Transceiver aperture	50 mm
**Transmit laser**	
Wavelength	1550 nm
Beam waist	17.1 mm
Power	11.7 dBm
**Machine vision**	
Focal length	500 mm
Field of view	$1.0^{\circ }\times 0.75^{\circ }$
Pixel scale	9 ${\upmu \hbox {rad/pixel}}$
Mount command rate	10 Hz
Beacon wavelength	532 nm
**Tip/tilt AO**	
Bandwidth	200Hz
Mirror actuation range	± 2 mrad
Mirror resolution	50 nrad

### Mount

Our deployable optical transceiver terminal was built around a PlaneWave Instruments L-350 precision altitude-azimuth astronomical mount. This mount provides smooth, accurate tracking and slew speeds of up to 50 deg/s for rapid acquisition. The mount is controlled by the remotely accessed single board computer located on the optical breadboard. Initial pointing of the mount can be provided by spacecraft two line element (TLE) ephemeris or aircraft automatic dependent surveillance-broadcast (ADS-B) retrieved over the internet, or in the case of this experiment, from GPS coordinates transmitted from the drone via 921.2 MHz LoRa signal. The vendor-provided mount API natively accepts TLEs and calculates the mount path accordingly, while ADS-B and GPS coordinates are converted into mount altitude and azimuth coordinates by our bespoke software layer above the mount API.

### Machine vision

**Figure 4:**
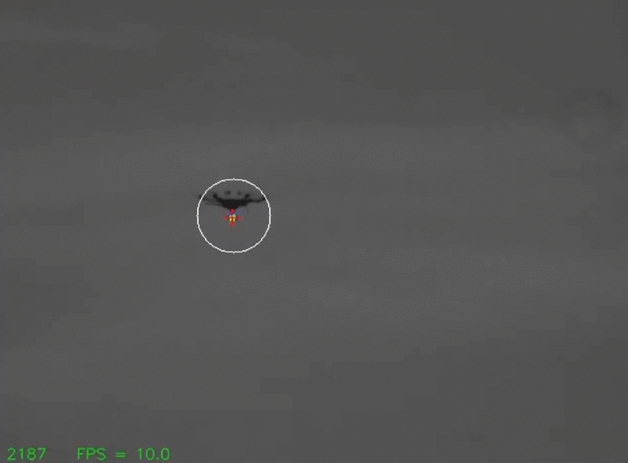
Image from the machine vision tracking camera. The red cross shows the detected position of the beacon LEDs. The white circle shows the tip/tilt mirror actuation range, centred on the predetermined hotspot.

GPS and TLEs are not sufficiently precise to point an optical ground station accurately enough to acquire its target. To provide an intermediate acquisition and tracking stage between TLE/GPS and the tip/tilt AO system, an MV system is used for optical closed loop control of the mount. We use a commercially available MV camera with an ${f=500}$ mm lens, giving a ${1.0^{\circ }\times 0.75^{\circ }}$ field of view. An example image is shown in Fig. 4. This is large enough to allow for errors in TLE/GPS-derived pointing, but with a fine 9 $\upmu$rad/pixel for tracking resolution. A broadband green filter was added to enhance the signal-to-noise ratio of the drone’s 532 nm beacon LEDs over the blue-sky background.

The response time of the mount limited the rate at which it could receive commands from the MV system to ${\sim \, 15}$ Hz, but to avoid intermittent CPU bottlenecks on the single board computer (SBC) we further limited the camera acquisition and command rates to 10 Hz. Each image was thresholded to detect the four beacon LEDs on the target that circumscribe the CCR. The pointing error is calculated from the pixel difference between the center of mass of the thresholded pixels and the “hotspot”; the pixel coordinates where the target must be located for the retroreflected laser to be coupled back into the SMF. The difference in pixel values are converted to errors in azimuth and altitude angles, and then fed to a software proportional, integral, derivative (PID) control loop to calculate offset rates, in arcseconds per second^25^, to apply to the mount to maintain the target on the hotspot. The hotspot is determined pre-flight using the 600 m and 2.4 km static links. The camera was aligned on the mount such that the *x*-axis aligned with azimuth, and the *y*-axis with altitude.

### Coherent communications

The 1550 nm laser communications signal under test was generated by a commercially-off-the-shelf C form-factor (CFP) 27.95 Gbaud dual polarization quadrature phase shift keying (DP-QPSK) digital coherent optics (DCO) module, with net line rate of 118.8 Gbps, typical of high-capacity optical fiber transport networks. This DCO module was controlled with an evaluation board, providing access to standardised pre-forward error correction (FEC) BER and received power measurements with a 1 s minimum performance monitoring interval^26^. This 1 s sampling rate is suitable for deployment in fixed fiber networks, but will not capture amplitude shifts seen on a free space link due to the shorter atmospheric coherence time (a few to tens of milliseconds). In-fiber commissioning of the DCO module demonstrated the threshold power corresponding to a FEC-correctable BER of $4.5\times 10^{-3}$ is approximately − 30 dBm (optional registers reporting optical signal-to-noise ratio^26^ were not implemented in this module). This BER value was taken to be the threshold for error-free communication, with the caveat that a 1 s period of reception averages out short duration bit error events over a free-space link and some instances may exceed the error-free threshold. Unfortunately, post-FEC BER nor QPSK constellations were available from this module.

As the DCO power measurements are taken at 1 Hz, fast power fluctuations due to atmospheric turbulence are subject to aliasing. Therefore, a 90:10 splitter sends 10% of the received light to a monitoring photodetector to capture received power information at 2 kHz. This sample rate is faster than the atmospheric coherence time, and allowed us to determine whether short duration deep fades due to turbulence or pointing errors were present; if signal is observed throughout the drone passes then the goal of robust SMF coupling is successful.

### Optics

The communications signal is fiber fed from the DCO module to the mount via an erbium-doped fiber amplifier (EDFA) nominally providing 20 dB gain and $<5$ dB noise. The amplified output power was verified using a handheld power meter before feeding to the mount, where it is transmitted from a fiber to free-space collimator as a beam of waist radius $w_0=1.14$ mm. The beam is directed to a 50:50 beamsplitter, needed for the tip/tilt AO correction of the returned beam, where 50% of the power is transmitted through the system. The transmitted portion of the beam is expanded by a $15\times$ Galilean beam expander (GBE) to a waist radius of $w_0=17.1$ mm from an aperture diameter of 50 mm. We chose this beam size to be as large as possible to minimise divergence due to diffraction, while remaining smaller than the expected worst-case Fried Parameter size ($r_0$, typically on the order of $\sim \, 10$ cm at 1550 nm for strong turbulence). In this regime, scintillation is negligible and first-order tip/tilt AO alone is sufficient to correct for atmospheric turbulence. The expanded beam is reflected off the piezo-electric tip/tilt mirror to a static launch mirror before exiting from the system. Note that in Fig. 1, these two mirrors are swapped for graphical convenience, but this is functionally identical.

After propagating across the atmospheric channel to the drone, the beam is retroreflected and returned to the transceiver where it follows the reverse path through the optics. This time, at the beamsplitter, the transmitted light is coupled back into the SMF to be sent to the communications module and monitoring photodetector, while the reflected light is focused onto a position sensitive quadrant photodetector (QPD). Variations in angle-of-arrival of the retroreflected beam imparted by turbulence and/or pointing errors are translated into lateral spot movement on the QPD, which is used by the PID loop and control electronics to drive the tip/tilt mirror actuation to maintain the spot centering.

The tip/tilt AO system consists of a two-inch diameter mirror mounted to a commercial fast Piezo tip/tilt platform and its associated electronics. The Piezo platform has a specified closed-loop angular resolution of 50 nrad and actuation range of $\pm \,2$ mrad in two dimensions. During this experiment, the tip/tilt loop was operated at 200 Hz. Due to atmospheric reciprocity^23^, the tip/tilt loop has the dual effect of correcting beam wander of the outgoing beam to maintain pointing, as well as correcting the angle-of-arrival of the return beam to maintain fiber-coupling efficiency.

A link budget for the experiment is presented in Table 2. The in-fiber transmit power was limited to a maximum of 11.7 dBm to avoid saturation of the QPD and mitigate the effect of prompt reflections, largely from the refractive elements of the beam expander. The terminal optics imposed a combined 15.7 dB loss across transmission and reception, leaving 26 dB of link margin above the - 30 dBm threshold for pointing, geometric, and atmospheric losses.

**Table 2:** Demonstration link budget.

Parameter	Value
In-fiber transmit power	11.7 dBm
Transmit beam split loss	− 3 dB
Geometric and clipping loss	− 1.7 dB
Receive beam split loss	− 3 dB
Single-mode fiber coupling loss	− 8 dB
Received power threshold for $10^{-4}$ BER	− 30 dBm
Link margin	26 dB

### Drone

**Figure 5:**
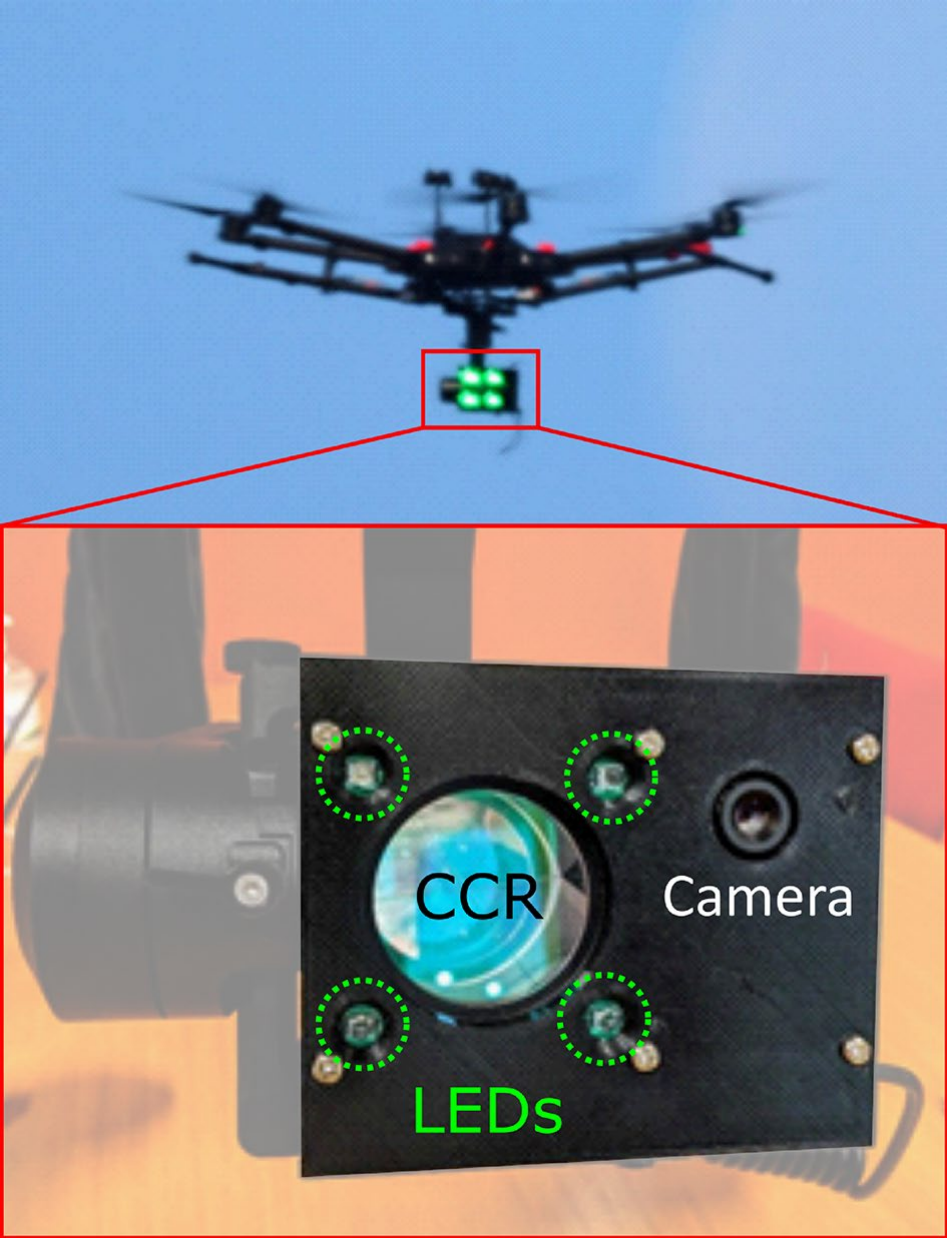
Top: the drone in operation, with MV beacon LEDs visible on the gimbal-mounted optical payload. Bottom: close-up of the optical payload showing the beacon LEDs, CCR, and camera.

To simulate the angular motion of a satellite in LEO, we use a professional grade drone carrying a gimbal-mounted optical payload consisting of a two-inch CCR to return the 1550 nm signal, four 532 nm beacon LEDs for MV tracking, and a camera for payload orientation. The drone also carries a GPS and barometric altimeter that relay coordinates to the optical terminal via LoRa for autonomous acquisition. The drone has a maximum horizontal velocity of 65 km/h, allowing us to easily mimic the ${\sim 1}$ deg/s angular tracking rates of LEO across the ${\sim \, 700}$ m distance to the optical terminal. Figure 5 shows the drone in flight with the payload LEDs illuminated (top), and a close up of the payload (bottom).

### Flight operations

After take-off, the drone climbed to a regulation-limited 120 m altitude and moved into position over the Swan River, then adjusted the gimbal pointing so the beacon LEDs were oriented toward the mount. The onboard GPS module continuously transmitted the drone’s position to the optical terminal computer, which was converted into altitude and azimuth angles to point the terminal at the drone. Once the LEDs were visible within the camera’s field-of-view, the MV loop was closed and the mount pointing adjusted to acquire and maintain the drone beacons on the hotspot.

**Figure 6:**
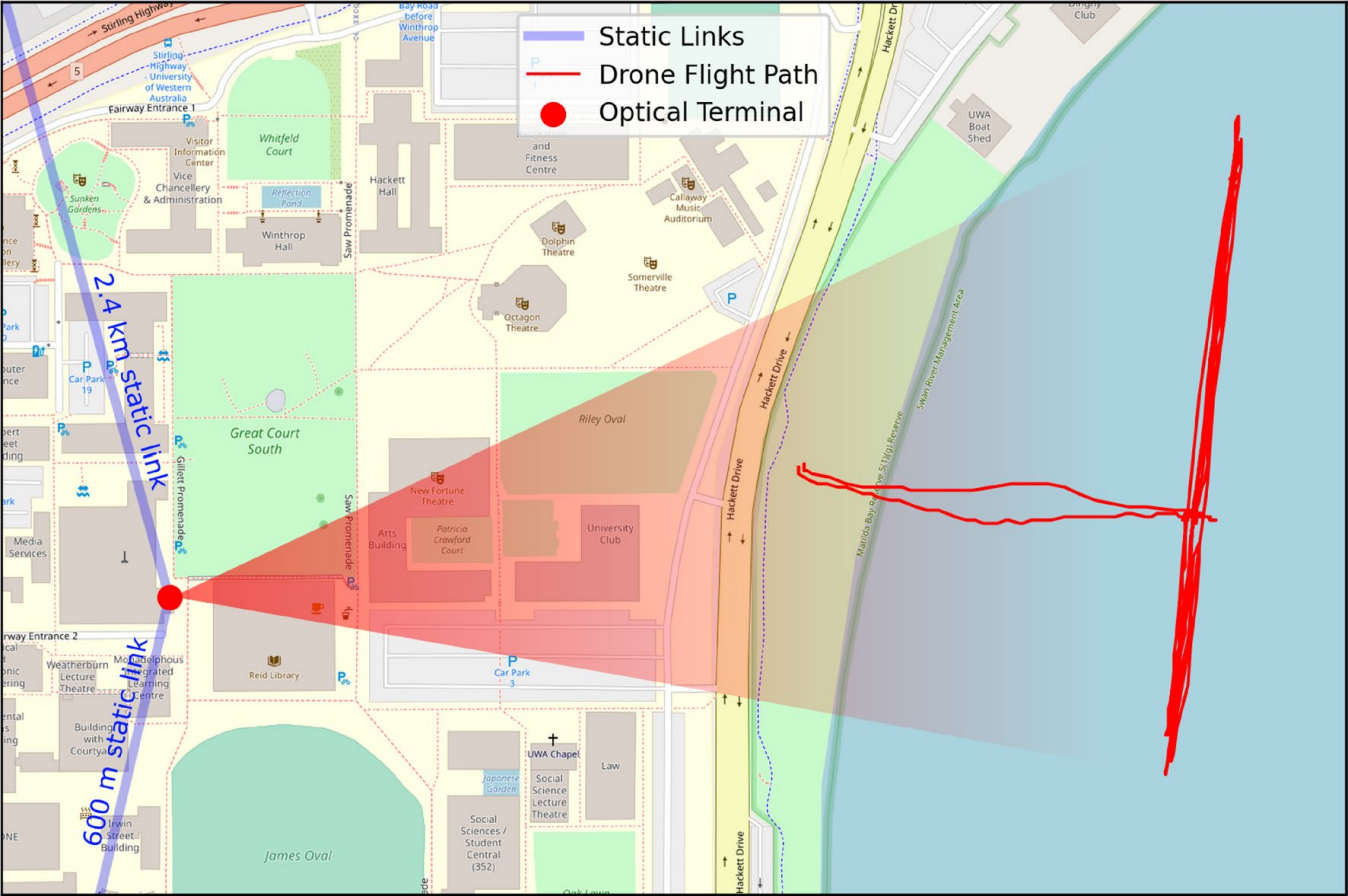
Map of flight area around the University of Western Australia campus in Perth, Western Australia. Red dot is the deployable optical terminal on the roof of the physics building, blue lines are static link paths, red line is the drone flight path during the 2022/04/21 flight. Map generated with OpenStreetMap data under the Open Database License (openstreetmap.org/copyright).

With the drone located on the MV hotspot, the laser was nominally incident on the CCR and signal was returned to the terminal. However, given the relatively short distance to the drone and its susceptibility to wind buffeting, return power was only intermittently observed until the tip/tilt loop was closed and signal was stable. The MV and tip/tilt loops ran concurrently to track the drone correcting for macroscopic motion, whether intentional or due to wind buffeting, as well as beam wander from atmospheric turbulence. The MV controlled the mount for high-amplitude, low-frequency ($\gtrsim \,1$ Hz) errors and the tip/tilt loop controlled the tip/tilt mirror for low-amplitude ($<2$ mrad), high-frequency errors. With both tracking loops closed, we flew the drone in passes replicating the tracking rates needed for free-space optical links to spacecraft in LEO. Figure 6 shows a map of the drone flight path, which was limited in the north by obstructed line-of-sight, and to the south by dense marine traffic.

## Results and discussion

**Figure 7:**
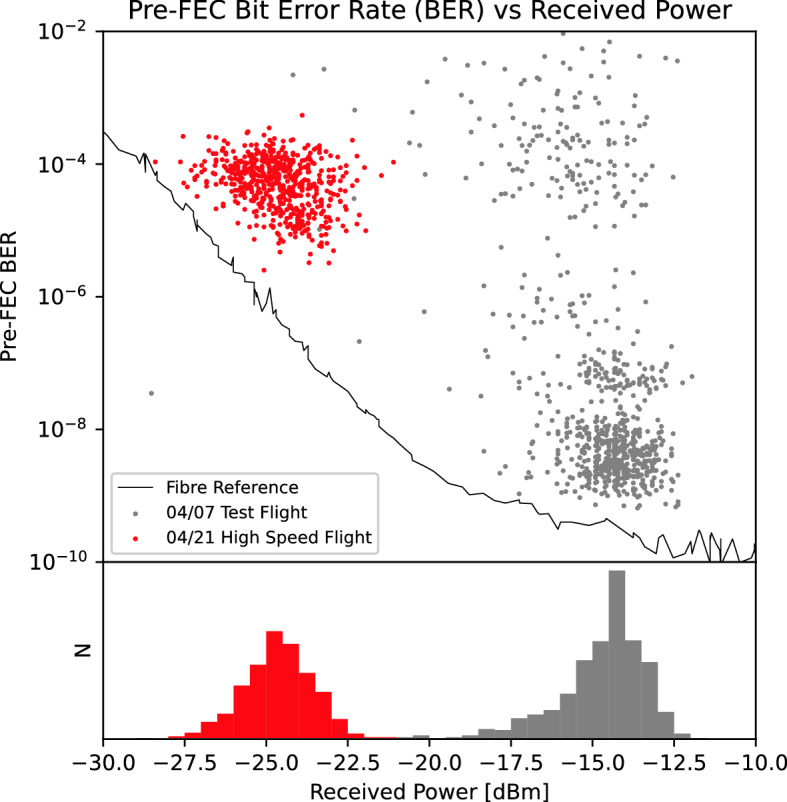
Top: pre-FEC BER versus received power. The black line is measurements taken in-fiber, delineating best possible performance. The gray dots are measurements from the 2022/04/07 test flights, red dots are from the 2022/04/21 high speed flight. Bottom: histogram of received power. Gray is from 2022/04/07 test flights, red is from 2022/04/21 high speed flight.

**Figure 8:**
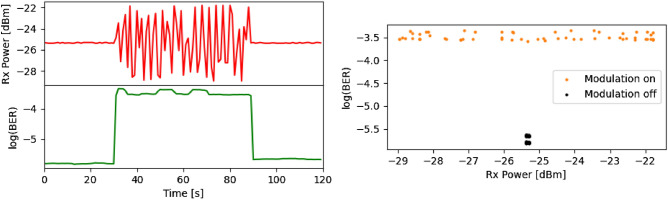
Left: time series for power (red) and log(BER) (green) for rapid power modulation test of DCO module. Right: scatter plot of BER versus power for modulation on (orange) and off (black).

Flights were conducted on 2022/04/07 and 2022/04/21, with the former being a series of test flights and the latter being the culminating “high speed” flight. Atmospheric conditions for these dates are shown in Table 3. Figure 7 shows the BERs versus received optical power (top panel) for the flights with in-fiber measurements for reference, as well as histograms for the received power (bottom panel) for both days. During testing the received optical power ranged from $-20$ to $-12$ dBm, implying total pointing, atmospheric, and other losses of 8–16 dB. However, on 2022/04/21 smoke was present due to controlled burns by the Parks and Wildlife Service; air quality monitoring from sites 16 km north and 18 km north-east reported peak PM$_{2.5}$ densities over 150 and 200 $\upmu$g/m$^3$ respectively, compared with average values for those sites of $\sim \, 20$ $\upmu$g/m$^3$. The increased density of micron-sized particulates imposed an apparent additional loss of $\sim \,10$ dB due to Mie scattering of the 1550 nm beam across the link, compared with the test measurements taken in the clearer PM$_{2.5}$ conditions on 2022/04/07. This reduced power resulted in an associated increase in BER. The measurements deviate from the in-fiber reference due to aliasing of the turbulence-induced power fluctuations occurring faster than the 1 Hz sampling rate. In-fiber testing with a signal modulated by $\pm \,3$ dB at 220 Hz produced a two orders of magnitude increase in BER, with the aliased power measurements distributed near uniformly across the modulated range, as seen in Fig. 8. We therefore conclude the clustering of points at the top right of Fig. 7 is due to a period of particularly high turbulent variability across the link.

**Table 3:** Atmospheric conditions in Perth for flight dates.

Parameter	2022/04/07	2022/04/21
Temperature ($^{\circ }$C)	30	25
Relative humidity (%)	25	34
Pressure (hPa)	1014	1023
Wind speed (km/h)	15	17
Wind direction	W	N
Cloud cover (%)	18	10
**Air quality (peak PM**_**2.5**_**, µg/m**^**3**^)		
Duncraig (16 km N)	$<10$	160
Caversham (18 km NE)	10	$>200$

**Figure 9:**
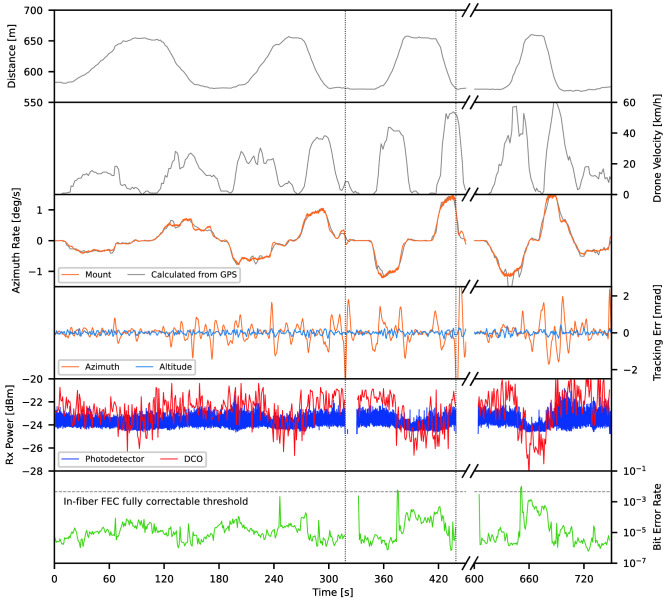
Time series data. From top to bottom: line-of-sight distance between the deployable optical terminal and drone as calculated from GPS. Drone horizontal velocity, as calculated from GPS. Mount azimuth tracking rate, as calculated from mount (orange) and GPS (grey). Machine vision tracking errors in azimuth (orange) and altitude (blue). Received optical power from photodetector (blue) and DCO module (red). BER. Times of link dropout are delineated with a dotted line ($t=318$ s and $t=439$ s).

Figure 9 shows time-series data for the 2022/04/21 high speed flight, showing drone-terminal distance, drone velocity, mount azimuth tracking rate, MV tracking error, received optical power (from DCO module and photodetector), and pre-FEC BER. Drone distance and velocity were calculated from GPS coordinates, which also provide an independent measure of the mount azimuth tracking rate in addition to the values reported directly by the mount. The MV tracking errors are the offsets in milliradians between observed drone position and the hotspot.

The measurement series spans a total of eight drone passes over ${\sim \, 750}$ s, with the longest uninterrupted link period of ${\sim \, 318}$ s, or four drone passes. The link is only broken at the end of the fourth and sixth passes (${t=318}$ s and ${t=439}$ s) when the drone decelerated abruptly at the flight area boundary, resulting in pointing errors too fast for the MV and too large for tip/tilt loops. When this occurred, the transmitted beam was no longer incident upon the CCR, and therefore no signal was returned; it is not the result of turbulence or poor link quality. During these periods of interruption, the MV tracking remains active as long as the drone remains visible in the ${1.0^{\circ }\times 0.75^{\circ }}$ field of view. When the tracking returns the drone to the hotspot, the tip/tilt loop is closed again.

During the flight, the drone ranged from ${\sim \, 550}$  to ${\sim \, 660}$ m line-of-sight distance. The drone reached a maximum speed of 60 km/h, corresponding to an azimuth tracking rate of 1.5 deg/s. The MV tracking errors show the effect of wind buffeting, with the azimuth error frequently spiking well above 1 mrad while the altitude error remains stable below 0.25 mrad.

The returned optical power and BER plots show the link was lost only when the MV error exceeded 2.5 mrad during the drone deceleration; somewhat greater than the manufacturer specified 2 mrad actuation limit of the tip/tilt mirror. The pre-FEC BER fluctuates between $\sim 10^{-6}$ and $\sim 10^{-3}$. Given the millisecond scale of turbulence-induced atmospheric coherence time, instances of high BER will dominate the average within each 1 s sample. Therefore, the BER floor is very likely pessimistic compared to what would be observed with shorter and more frequent sample periods, which conversely would likely resolve more spikes above the FEC-correctable threshold. However, we can infer that for a practical communications link, when the pre-FEC BER approached and exceeded the (fiber-verified) FEC-correctable threshold of $4.5\times 10^{-3}$, reliable communication could still be established with an appropriate retransmission protocol at the data layer. With this in mind, the results serve as confirmation that robust data transmission was maintained throughout the flight. Given the presence of smoke induced power loss and the limitations of the DCO for free-space links, the important result is the maintaining of the SMF coupling, rather than the specific BER behaviour.

The monitoring photodetector, receiving only 10% of the returned signal, was operating near the lower limit of its dynamic range, where its response is non-linear making measurements less accurate than the DCO reported values. The values shown in Fig. 9 were shifted by + 10.7 dB to account for the splitter and normalize the output to the DCO measurements. Due to the non-linearity it does not show the same variation in power as the DCO aliased measurements, but its 2 kHz sampling rate serves the critical purpose of showing that power fades are not occurring on timescales shorter than the 1 s DCO sample time; verifying that we maintained fiber coupling throughout the drone passes despite atmospheric turbulence, wind buffeting, and high angular velocity of the drone. To the best of our knowledge, this is the first such demonstration of tip/tilt AO-stabilised robust SMF coupling at the angular tracking rates needed for coherent ground-to-LEO links.

It is not possible to quantify the turbulence strength observed across the drone link from the data available as the power measurements are post-tip/tilt correction and any angle-of-arrival variations from turbulence are coupled with those from drone movement/vibration. Measurements without tip/tilt for comparison were also not possible as the tip/tilt loop was required to keep the beam on the CCR in the presence of wind buffeting. However, given the fact that tip/tilt was sufficient to keep the beam centered on the QPD, we can conclude that scintillation was negligible and the integrated turbulence resulted in a Fried parameter size $r_0$ larger than the receiver diameter of 50 mm. For the round trip link distance of 1.2 km at 1550 nm, this would correspond to a upper bound $C_n^2$ of $~5\times 10^{-14}$ m$^{-2/3}$ throughout the experiment.

In some respects, a low-altitude drone link is more challenging than a link to a spacecraft. At the $\sim \, 600$ m link length, the change in beam size due to divergence is negligible such that at the drone it is still only on the order of the CCR size. The drone was subjected to wind buffeting, causing motion at the scale of tens of centimeters even in mild winds and moving the CCR in and out of the beam when the tip/tilt AO loop was not closed. This motion was faster than the MV could correct the mount pointing, meaning that the tip/tilt AO system was correcting for this in addition to angle-of-arrival variations due to atmospheric turbulence. Furthermore, without a TLE spacecraft ephemeris providing an a priori coarse tracking path, the MV was responsible for tracking of the drone’s large scale motion rather than making minor corrections to a pre-defined path. Despite these challenges the terminal maintained the link, with dropouts occurring only during abrupt deceleration of the drone as it approached flight boundaries, where the correction required was too rapid for the mount tracking and too large for the tip/tilt mirror. This situation is not analogous to any practical scenario of ground-to-LEO communications with a cooperative target.

A further drawback of the airspace and altitude restrictions on the drone was that tests were limited to tangential paths at a distance of $>550$ m. In this situation, the angular velocity is almost purely azimuthal, at a low altitude angle ($\sim \, 8^{\circ }$). The mechanical azimuth rate of the mount needed to track a target on sky with a given angular velocity scales inversely with the cosine of the altitude angle, so the closer to zenith a satellite transits, the faster the azimuth axis must rotate. A more robust test would be a flight that transits close to zenith, more closely approximating the tracking demands on the mount axes where the required azimuth rate increases dramatically. We aim to address this in future experiments with light aircraft.

For this work we used the retroreflected beam as its own tip/tilt beacon, which provides an angle-of-arrival error signal despite atmospheric reciprocity due to the truncation of the Gaussian beam at the CCR on the drone payload^22^. This was convenient as it minimised the size and weight requirements of the drone. For a real LEO downlink, the spacecraft would either transmit a dedicated beacon signal at a separate wavelength from the data signal, or a portion of the data signal could be siphoned to use as the tip/tilt (or higher order AO) beacon. In either scenario, the operation of the tip/tilt loop remains unchanged from this work.

Our terminal has demonstrated the tracking capability for maintaining coherent ground-to-LEO communications, but to develop the terminal into a system capable of real uplink and downlink to LEO requires some optimizations. The line-of-sight velocity of the drone in this experiment produced a Doppler shift of at most $\sim \, 1$ MHz, whereas the Doppler shift seen from a spacecraft at 500 km altitude LEO is of order $\pm \,10$ GHz during each orbital pass. For this experiment Doppler shift was negligible compared to the $\pm \,1.8$ GHz accuracy of the DCO module, but for coherent communications from LEO, a local oscillator capable of sweeping across a $\sim \,20$ GHz frequency range would be required.

The MV system would also need to be tailored to the beacon being used by the spacecraft. The limiting factor for our drone flights were the positional uncertainty from the GPS at a relatively short distance, requiring a large field of view. A simple lens and visible camera were sufficient as the beacon LEDs provided ample signal. A spacecraft beacon would be significantly fainter and therefore the MV system might be incorporated within the larger receiver optics using a dichroic/other beamsplitter to increase sensitivity and resolution. Depending on field-of-view constraints, separate coarse and fine MV systems may be needed^25^. It is possible the downlink signal itself could serve as both tip/tilt and MV beacons, which would require a camera sensitive at the signal wavelength. The demands placed on the MV for LEO would also be significantly less compared to the wind-buffeted drone, meaning the control loop could operate at a slower rate and allow for longer integration time of the fainter beacon.

An increase in receiver aperture diameter is needed for more collecting power of the downlink signal, and an increase in transmitter aperture is needed to reduce beam divergence and geometric losses over the link distance to LEO. Aperture sizes of a few tens of centimeters are sufficient for ground-to-LEO links^25,27,28^. If the ratio of aperture size to Fried Parameter $D/r_0$ is greater than one, either due to large aperture or strong turbulence, higher order correction beyond tip/tilt is required to efficiently couple into SMF. This can be accomplished with traditional AO^15,29^, or with novel “passive” methods such as photonic lanterns^30^ or multi-plane light conversion^31^. In combination with atmospheric phase-stabilisation technology^32–34^, such a deployable optical terminal could even facilitate secure ground-to-LEO continuous variable quantum key distribution (CV-QKD)^35^.

## Conclusion

We have demonstrated a robust, high speed coherent free-space optical communications link between a deployable optical terminal and drone moving at LEO-like angular velocities. Combining MV optical tracking and large actuation range tip/tilt AO, we maintained transmitted beam pointing and retroreflected beam angle-of-arrival in the presence of atmospheric turbulence and macroscopic motion to sustain the 100 Gbps link. Single mode fiber coupling is requisite for high capacity coherent communications, and ground stations with capabilities such as described here will relieve the data bottleneck between earth and LEO and provide ubiquitous internet-like speeds to space.

## Data Availability

The datasets used and/or analysed during the current study available from the corresponding author on reasonable request.
